# Microglia activation in a model of retinal degeneration and TUDCA neuroprotective effects

**DOI:** 10.1186/s12974-014-0186-3

**Published:** 2014-10-29

**Authors:** Agustina Noailles, Laura Fernández-Sánchez, Pedro Lax, Nicolás Cuenca

**Affiliations:** Physiology, Genetics and Microbiology, University of Alicante, San Vicente University Campus, E-03080 Alicante, Spain; Multidisciplinary Institute for Environmental Studies ‘Ramon Margalef’, University of Alicante, Alicante, Spain

**Keywords:** Glia, Retinitis pigmentosa, Neuroprotection, Confocal microscopy

## Abstract

**Background:**

Retinitis pigmentosa is a heterogeneous group of inherited neurodegenerative retinal disorders characterized by a progressive peripheral vision loss and night vision difficulties, subsequently leading to central vision impairment. Chronic microglia activation is associated with various neurodegenerative diseases including retinitis pigmentosa. The objective of this study was to quantify microglia activation in the retina of P23H rats, an animal model of retinitis pigmentosa, and to evaluate the therapeutic effects of TUDCA (tauroursodeoxycholic acid), which has been described as a neuroprotective compound.

**Methods:**

For this study, homozygous P23H line 3 and Sprague-Dawley (SD) rats were injected weekly with TUDCA (500 mg/kg, ip) or vehicle (saline) from 20 days to 4 months old. Vertical retinal sections and whole-mount retinas were immunostained for specific markers of microglial cells (anti-CD11b, anti-Iba1 and anti-MHC-II). Microglial cell morphology was analyzed and the number of retinal microglial was quantified.

**Results:**

Microglial cells in the SD rat retinas were arranged in regular mosaics homogenously distributed within the plexiform and ganglion cell layers. In the P23H rat retina, microglial cells increased in number in all layers compared with control SD rat retinas, preserving the regular mosaic distribution. In addition, a large number of amoeboid CD11b-positive cells were observed in the P23H rat retina, even in the subretinal space. Retinas of TUDCA-treated P23H animals exhibited lower microglial cell number in all layers and absence of microglial cells in the subretinal space.

**Conclusions:**

These results report novel TUDCA anti-inflammatory actions, with potential therapeutic implications for neurodegenerative diseases, including retinitis pigmentosa.

## Background

Retinitis pigmentosa (RP) is a type of hereditary retinal degeneration with high levels of clinical and genetic heterogeneity. This neurodegenerative retinal disorder is characterized by a primary degeneration of the photoreceptor rods causing peripheral vision loss and night blindness. With the progression of the disease only the cone cells of the fovea remain functional, leading to a classical tunnel vision. In the end stage of the disease with the dysfunction of all cone cells, the central visual field degenerates and leads to a complete blindness. It is currently known that more than 100 different mutations in the rhodopsin-encoding gene (*RHO*) are related with 30 to 40% of autosomal dominant cases. The Pro-23-His mutation is the most prevalent cause of RP [1], being the genetic cause of about 15% of autosomal dominant RP cases in the United States [2]. It is currently known that the P23H mutation causes misfolding and retention of rhodopsin in the endoplasmic reticulum [3]. Others studies suggest that the mechanism of RP involves cellular stress, inflammatory response, retinal remodeling, and the final common pathway of programmed photoreceptor cell death or apoptosis [4-6]. Similar mechanisms were involved in other retinal degenerations like glaucoma, diabetic retinopathy or macular degeneration [6].

Microglial cells act as the resident immune cells of the central nervous system (CNS), including the retina, playing the role of primary mediators of inflammation. In the absence of disease, microglial cells exhibit a ramified morphology with a small, round soma, and various branching processes and play critical functions in axonal growth, synaptic remodeling and neuronal survival via phagocytosis of cellular debris and relief of a variety of cell signaling factors [7,8]. In response to negative stimulus, tissue injury or free radicals, microglial cells assume a reactive state, characterized by a shortening and widening of microglial processes [9]. These reactive microglial cells can progress into phagocytic microglial cells. In this amoeboid form, microglial cells lack cellular processes and perform characteristic macrophage functions. Activated microglial cells have been observed in mouse models of autosomal recessive RP [10,11], in *rds* mice [12] and in rat models of inherited retinal degeneration, including Royal College of Surgeons rats [13].

Activated microglial cells are able to generate trophic biomolecules, glutamate transporters and antioxidants that promote the correct neuronal functioning. But, likewise, activated microglial cells are capable of producing potentially neurotoxic substances such as nitric oxide (NO) and pro-inflammatory cytokines (IL-1α, IL-1β, TNF-α, IFN-γ, IL-6, and so on) that are involved in neurological diseases and CNS disturbances, like infections or chemical damage and aging [14-17].

In retinal neurodegenerative diseases, chronic microglial activation and neuroinflammation are common phenomena. In RP, the primary death of rod photoreceptors triggers the activation of microglial cells and their migration to the outer retina to eliminate cellular debris. It has been proposed that these activated microglial cells may release cytotoxic factors such as NO that kills adjacent photoreceptors, including cones [18]. In age-related macular degeneration, previous studies show that microglial cells become pathogenic with age, causing a chronic activation that will influence the health of retinal tissue [19,20]. Microglial cells also play a critical role in the progression of glaucoma. Many studies show that the number, morphology, distribution and antigen-presenting activity of microglial cells change in glaucomatous eyes highlighting their importance in the pathological process [21-23]. In experimental models of diabetic retinopathy, microglial cells also appear altered suggesting that the activation process is underway. However, it is actually unknown what degree of this activation is due to resident microglial cells of the retina or to circulating monocytic cells [24].

Due to this duality, the function(s) performed by microglia in regulation of injured neurons remain uncertain. Numerous studies suggest that microglial activation is harmful for neuronal survival, showing that the inhibition of microglial activation and cytokine secretion causes a reduction of neuronal loss [25,26]. However, other research makes evident the neuroprotective effect of microglial activation [27,28]. Some research support that the trophic and toxic effectors in microglia are controlled differentially depending on the severity of neuronal lesion [29]. Under pathological conditions, microglial cells of the retina are subjected to various kinds of endogenous and exogenous signals. These stimuli trigger local proliferation and changes in shape and morphology. Also, microglial cells alter their location in the retinal tissue, cytokine release pattern and expression of surface molecular markers. These characteristic immunological reactions and the absence/failure of the self-regulation engine may lead to an increase of retinal damage and pro-apoptotic events [10,18,30].

In this study, we address the hypothesis that the neuroprotective compound, tauroursodeoxycholic acid (TUDCA), is able to prevent microglial activation, modify its expression pattern and delay the photoreceptor cells loss in an animal model of RP. We have employed in our study, P23H and Sprague-Dawley (SD) rats to assess the therapeutic potential of TUDCA on photoreceptor degeneration and functional activity of the retina in these animal groups.

## Methods

### Animals

Homozygous P23H line 3 rats, obtained from Matthew LaVail [31], were used in this study as a model of RP. Age-matched wild-type SD rats (Harlan, IN, USA) were used as control. All animals were housed in cages under controlled photoperiod (12 hours light/12 hours dark), temperature (23°C ±1°C) and humidity (55 to 60%). Food and water were available *ad libitum*. All animals were handled in accordance with current regulations for the use of laboratory animals (NIH, ARVO and European Directive 2010/63/UE) in order to minimize animal suffering and limit the numbers used for the experiments. The study had the approval of the Research Ethics Committee of the University of Alicante.

### TUDCA treatment

TUDCA, purchased from Calbiochem (Gibbstown, NJ, USA), was dissolved in physiological saline (0.9% NaCl) just before administration by using an ultrasonic bath to avoid bubble formation. TUDCA was administered weekly to P23H line 3 rats (n = 6) at 500 mg/kg (intraperitoneally, ip) from P (postnatal day) 20 to P120, when these animals can be considered to have undergone extensive retinal degeneration [31-34]. Untreated P23H rats (n = 6) received the same volume of saline at the same time points. In order to adjust the amount of TUDCA and vehicle administered, the animal body weight was measured before each drug injection. Likewise, SD rats (n = 4) received a weekly injection of saline according to their weight.

### Retinal immunohistochemistry

Histological studies of the retinas were performed at P120. Cryostat vertical sections and retinal whole-mounts were obtained and processed for immunolabeling following well established procedures [35-38]. Animals were sacrificed in the morning by administration of a lethal dose of pentobarbital. After marking the dorsal margin of the limbus with a suture, eyes were enucleated and fixed in 4% (w/v) paraformaldehyde during 1 hour at room temperature (RT). After being washed in 0.1 M phosphate buffer pH 7.4 (PB), eyes were cryoprotected sequentially in 15, 20 and 30% sucrose. The cornea, lens and vitreous body were removed, and the eyecups were processed for vertical sections and whole-mounts.

For whole-mounts immunohistochemistry we utilized immunoperoxidase labeling. After fixation, retinas were dissected out from choroid and flat-mounted on a nitrocellulose filter, with the photoreceptor layer side up. Endogenous peroxidase activity was suppressed by immersion in 1% H_2_O_2_ (Sigma, St. Louis, MO, USA) in PB (10 minutes, RT). In order to break aldehyde bonds and enhance the permeability of the tissue, the retinas were incubated first in 2.28% sodium m-periodate (Sigma, St. Louis, MO, USA) in PB (5 minutes, RT) and then in 0.02% sodium borohydride (Panreac, Barcelona, Spain) in PB (5 minutes, RT). After a blocking step (10% normal goat serum in PB plus 0.5% triton X-100 for 1 hour), retinas were incubated for 3 days at 4°C under agitation with the primary antibody: mouse anti-rat integrin alpha M (CD11b) clone OX-42 (1:500; Chemicon, Temeluca, CA, USA), Retinas were washed 4 times in PB (5 minutes, RT) and then incubated for 1 day at 4°C in biotinylated goat anti-rabbit secondary IgG antibody at 1:100 dilution in PB plus 0.5% triton X-100. The retinas were washed before transferring to a solution of avidin-biotin-peroxidase complex (ABC) (Elite ABC kit, Vector Laboratories Ltd, Cambridgeshire, UK) in PB containing 0.5% triton X-100 for 1 day. Finally, the retinas were washed in PB and pre-incubated under agitation in the dark with 3,3′-diaminobenzidine tetrahydrochloride (DAB, Sigma, St. Louis, MO, USA; 0.5 mg/ml in PB) for 15 minutes and further incubated with fresh DAB solution with 0.033% H_2_O_2_ and 0.025% ammonium nickel (II) sulfate hexahydrate (Sigma, St. Louis, MO, USA). Washing with distilled water stopped the DAB reaction. Whole retinas were flat-mounted in Citifluor (Citifluor Ltd; London, UK) with the ganglion layer side up, and coverslipped for optical microscopy viewing on a Leica DMR microscope (Leica Microsystems, Wetzlar, Germany).

For immunofluorescence imaging, vertical sections were made. Eyecups were embedded in Tissue-Tek OCT (Sakura Finetek, Zoeterwouden, Netherlands) and frozen in liquid N_2_. Sixteen-micrometer-thick sections were obtained at −25°C, mounted on slides (Superfrost Plus; Menzel GmbH and Co. KG, Braunschweig, Germany) and air-dried. Before further use, slides were thawed and washed 3 times in PB and treated with blocking solution (10% donkey serum, 0.5% triton X-100 in PB) for 1 hour. Sections were subjected overnight at room temperature to single or double immunostaining with the primary antibodies: polyclonal rabbit anti-ionized calcium-binding adapter molecule 1 (Iba1, 1:1,000; Wako Chemicals, Richmond, VA, USA) and mouse anti-major histocompatibility complex (MHC) class II RT1B clone OX-6 (1:200, AbD Serotec, Kidlington, UK), diluted in PB plus 0.5% triton X-100. The secondary antibodies used were donkey anti-mouse or anti-rabbit IgG conjugated to Alexa Fluor 488 or 555 (Molecular Probes, Eugene, OR, USA) at a 1:100 dilution. Images were finally obtained under a Leica (Wetzlar, Germany) TCS SP2 confocal laser-scanning microscope. The spatial resolution employed was 1,024 × 1,024, the pinhole was set at 1 airy units (AU) and Z stacks were made with 15 pictures (1.5 μm steps). All stacks were collected using a x40 objective and the acquisition rate was 16 frames per second. Final images from SD and P23H groups were processed in parallel using Adobe Photoshop 10 software (Adobe Systems Inc., San Jose, CA, USA). A subset of vertical sections was stained by immunoperoxidase labeling.

### Analysis of microglia cell number and morphology

Morphology, spatial distribution and mean number of microglial cells were analyzed in the inner and outer plexiform layers (IPL, OPL), the ganglion cell layer (GCL) and the subretinal space (SS) of whole-mount retinas. Macrophages detected in the GCL during the analysis were also accounted. Four to six retinas from each animal group (TUDCA-treated, untreated and SD animals) were examined. In each retina, 12 representative regions of 0.227 mm^2^ each were analyzed, 6 regions equidistantly arranged on the superior-inferior axis of the retina and 6 fields disposed on the temporal-nasal axis; thus sampling representative peripheral, medial and central areas of the superior, inferior, temporal and nasal quadrants of each retina. In each of the 12 regions analyzed in each retina, every one of the cell bodies labeled with immunoperoxidase in each of the 3 layers analyzed was manually traced using a camera lucida attached to the Leica DMR microscope (Leica Microsystems, Wetzlar, Germany). Each retinal layer was determined according to the vascular stratification of the retinal tissue. The images created were subsequently digitized using the image-editing software Photoshop (Adobe Systems Inc., San Jose, CA, USA). The distribution pattern of microglial cells in each retinal layer was assessed by measuring the distances to the nearest neighbors of each microglial cell using ImageJ software [39]. The distances to the nearest neighbors were classified in histograms, statistically analyzed and compared with a nearest neighbor analysis of a random pattern of the same density and standard deviation [39,40]. For nearest neighbor distance analysis we used images collected from the medial area of the retina (superior quadrant).

**Figure 1:**
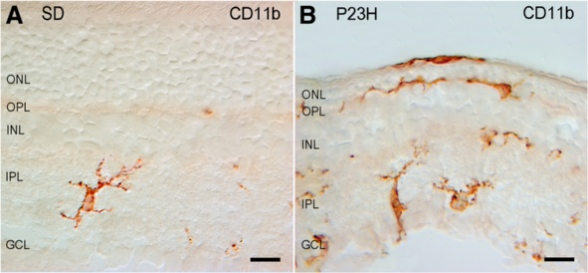
**Distribution and morphology of microglial cells in Sprague-Dawley (SD) (A) and P23H (B) rats.** Retinal vertical sections were immunolabeled with CD11b (OX-42). Note the presence of amoeboid CD11b-positive cells in different layers of the P23H rat retina, including the subretinal space. GCL, ganglion cell layer; IPL, inner plexiform layer; INL, inner nuclear layer; OPL, outer plexiform layer; ONL, outer nuclear layer. Scale bar: 10 μm.

**Figure 2:**
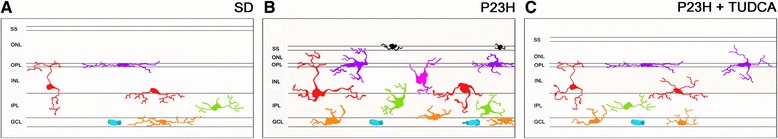
**Drawing of the most representative morphology and location of microglial cells in Sprague-Dawley (SD) (A), untreated P23H (B) and tauroursodeoxycholic acid (TUDCA)-treated P23H (C) rats.** Note the absence of microglial cells into the subretinal space of P23H rats treated with TUDCA. GCL, ganglion cell layer; IPL, inner plexiform layer; INL, inner nuclear layer; OPL, outer plexiform layer; ONL, outer nuclear layer; SS, subretinal space.

**Figure 3:**
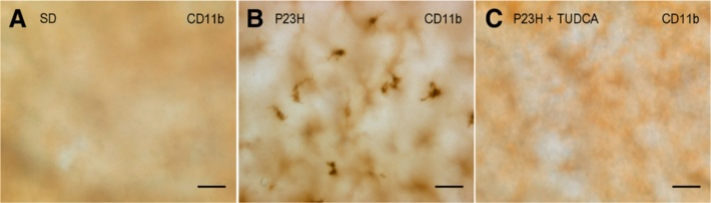
**Migration of microglia into the subretinal space of untreated P23H rats.** Whole-mount retina of a SD **(A)**, untreated P23H **(B)** and tauroursodeoxycholic acid (TUDCA)-treated P23H **(C)** rat immunolabeled with CD11b (OX-42), showing the presence of amoeboid microglial cells in the subretinal space of untreated P23H rats. Scale bar: 40 μm.

**Figure 4:**
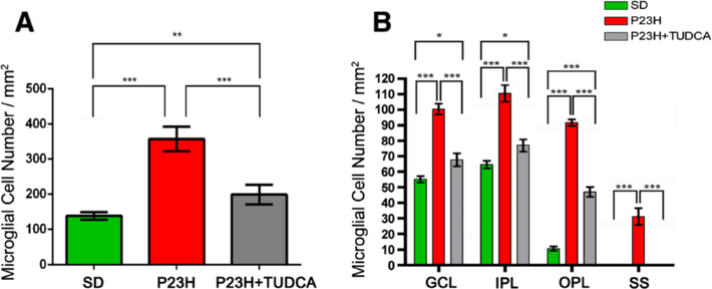
**Quantification of retinal microglial cells. (A)** Average number of positively stained microglial cells quantified in whole-mount retinas from SD (n = 4; green), untreated P23H (n = 6; red) and tauroursodeoxycholic acid (TUDCA)-treated P23H rats (n = 6; grey). **(B)** Average number of microglial cells in the GCL, IPL, OPL and SS of the retinas analyzed in **(A)**. *P <0.05, **P <0.01, ***P <0.001; ANOVA, Bonferroni’s test.

To analyze microglial activation in each animal group, we used cryostat vertical retinal sections immunostained with Iba1 and MHC-II. We examined three vertical sections non-consecutive per animal and we studied six animals per experimental group. All the images analyzed were collected from the central area of the retina, close to the optic nerve. In every retinal section, the total number of microglial cells expressing one or both markers (Iba1^**+**^/MHC-II^**−**^, Iba1^**−**^/MHC-II^**+**^ and Iba1^**+**^/MHC-II^**+**^) was counted using light microscopy at x63 magnification. The obtained data were referred to the length of each retinal section, measured using the ImageJ software.

### Statistical analysis

Statistical analyses were performed using the GraphPad software from Prism (La Jolla, CA, USA), in order to evaluate differences between SD, P23H untreated and P23H TUDCA-treated rats. A one-way ANOVA was used to evaluate differences in the mean number of microglial cells, and a two-way ANOVA was performed to compare the number of microglial cells corresponding to each one of the three expression patterns analyzed (Iba1^**+**^/MHC-II^**−**^, Iba1^**−**^/MHC-II^**+**^ and Iba1^**+**^/MHC-II^**+**^). When a 0.05 level of significance was found*, post hoc* pairwise comparisons using Bonferroni’s test were performed. Normal distributions and homogeneity of variance were found for all analyzed categories. Values of *P* <0.05 were considered statistically significant. Data were plotted as the average ± standard error of the mean (SEM).

## Results

### Distribution and morphology of retinal microglia in SD and P23H rats

Microglial cells were identified by specific labeling with CD11b, a constitutive marker of microglia and macrophages. In normal SD rat retinas, microglial cells were distributed in a plexus located at the inner and outer plexiform and ganglion cell layers (Figure 1A). Microglia in SD retinas showed a tiny cell soma, little perinuclear cytoplasm, and a large number of fine, branched processes covered in numerous projections. In the ganglion cell layer, few CD11b-positive cells with amoeboid morphology were also found.

**Figure 5:**
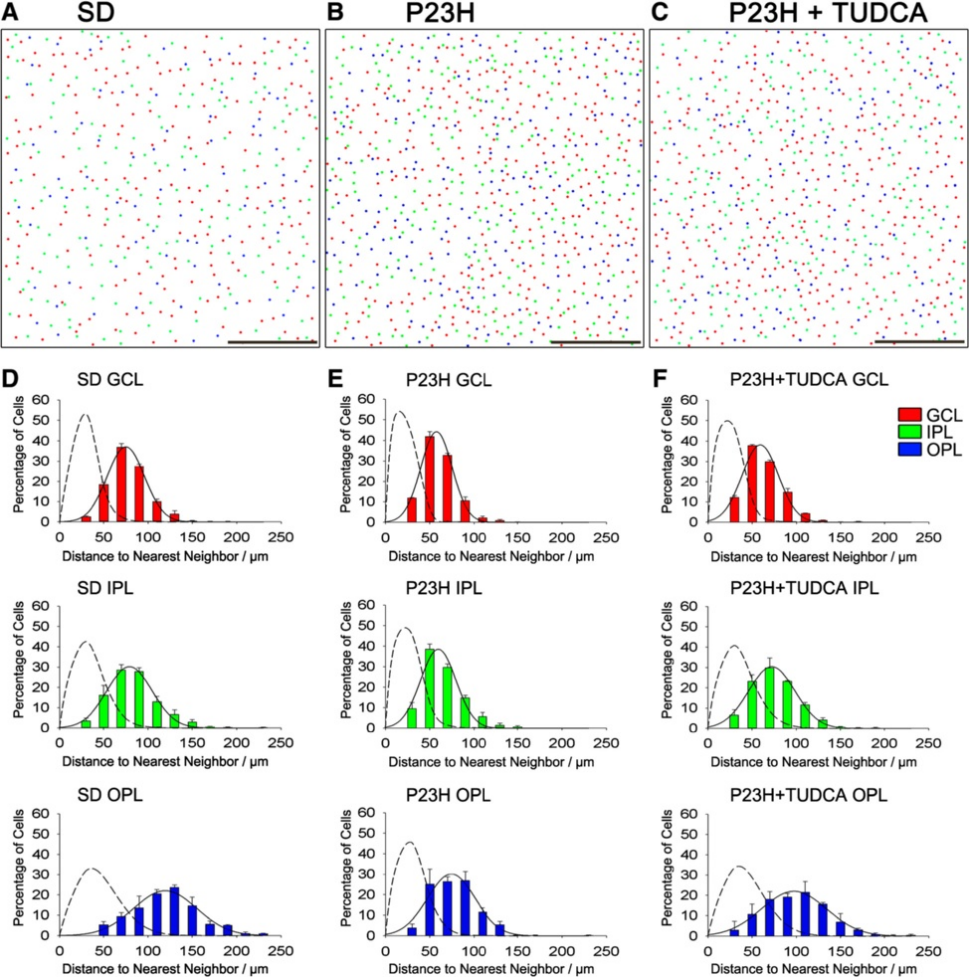
**Analysis of the distribution pattern of microglial cells. (A-C)** Drawings of microglial cell bodies labeled with immunoperoxidase in a representative area of the retina of a SD **(A)**, untreated P23H **(B)** and tauroursodeoxycholic acid (TUDCA)-treated P23H rat **(C)**. The localization of microglial cells in each of the retinal layers studied has been specified: GCL, red; IPL, green; OPL, blue. All images were collected from the medial area of the retina, in the superior quadrant. **(D-F)** Histograms of the nearest neighbors analysis in the OPL, IPL and GCL of SD **(D)**, untreated P23H **(E)** and TUDCA-treated P23H rats **(F)**. The solid line indicates the Gaussian function fitted to the data. The nearest neighbors analysis of a random pattern of the same density and standard deviation has been included for comparisons (dotted line). GCL, ganglion cell layer; IPL, inner plexiform layer; OPL, outer plexiform layer. Scale bar: 500 μm.

In the untreated P23H rat retina, an increase in microglial cell number was found compared with control SD rat retinas (Figure 1B). Moreover, numerous amoeboid CD11b-positive cells were observed in all retinal layers, with greater presence in IPL, OPL and the space that lies between the photoreceptors and the RPE, the SS. The most apparent morphologic features of amoeboid CD11b-positive cells were scarce short and thick primary and terminal processes and enlarged soma. In P23H rats treated with TUDCA, amoeboid CD11b-positive cells were less abundant than in untreated P23H rats in all layers of the retina, with absence of microglial cells into the SS. Also, the microglial morphology of P23H-treated group was most closely related with SD control group than with non-treated P23H group. Figure 2 shows a schematic representation of the distribution and morphology of microglial cells in SD, P23H-untreated and P23H TUDCA-treated rats.

**Figure 6:**
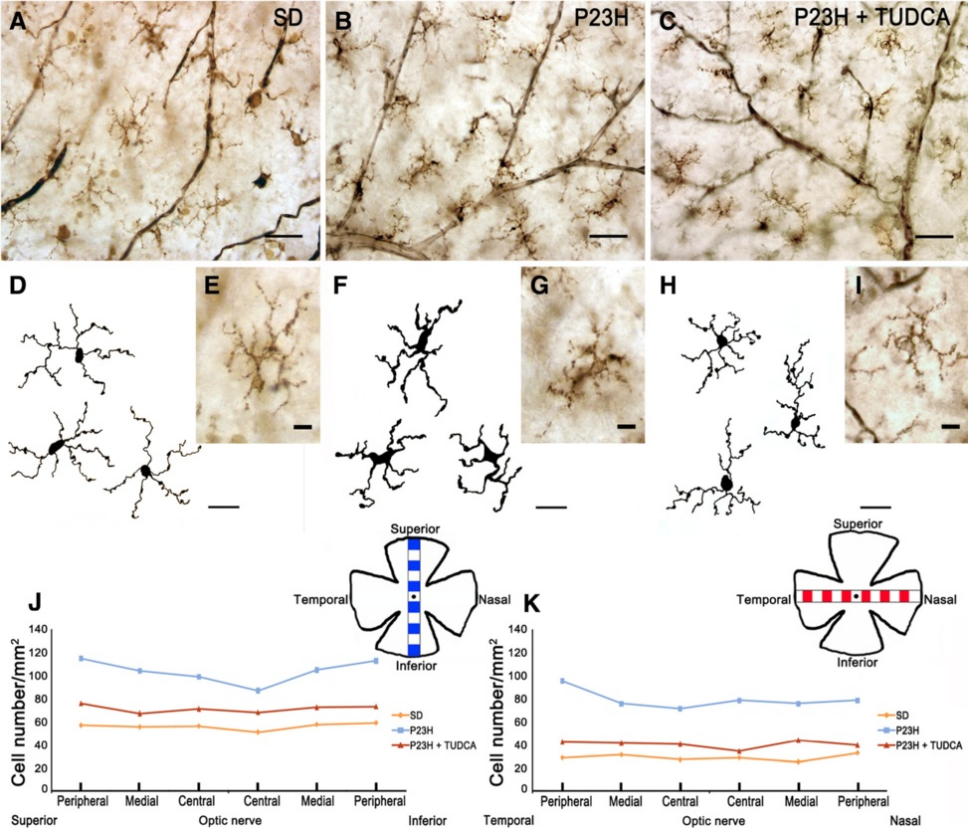
**Morphology, number and distribution of microglial cells in the ganglion cell layer (GCL). (A-** **C)** Representative images of whole-mount rat retinas from a SD **(A)**, untreated P23H **(B)** and tauroursodeoxycholic acid (TUDCA)-treated P23H rat **(C)** labeled with immunoperoxidase. **(E,** **G,** **I)** Magnifications of **(A)**, **(B)** and **(C)**, respectively. **(D,** **F,** **H)** Drawings of microglial cells from SD **(D)**, untreated P23H **(F)** and TUDCA-treated P23H rat **(H)**. **(J, K)** Microglial density, expressed as number of cells per mm^2^, in each of 12 representative regions in each retina: 6 equidistantly arranged on the superior-inferior axis of the retina **(J)** and 6 disposed on the temporal-nasal axis **(K)**. The scheme in the margin of each panel represents the position in the retina of each representative region analyzed. **(A, B, C)** scale bar: 40 μm; **(D, F, H)** scale bar: 20 μm; **(E, G, I)** scale bar: 10 μm.

Figure 3 illustrates the presence of microglia into the SS of untreated P23H rats (Figure 3B). Nearly all microglial cells found in this stratum showed morphologic features of amoeboid CD11b-positive cells. These microglial cells probably reach the retina from the sclera and are not resident microglia. These cells were completely nonexistent not only in normal retinas of SD rats (Figure 3A), but also in TUDCA-treated P23H rats (Figure 3C).

**Figure 7:**
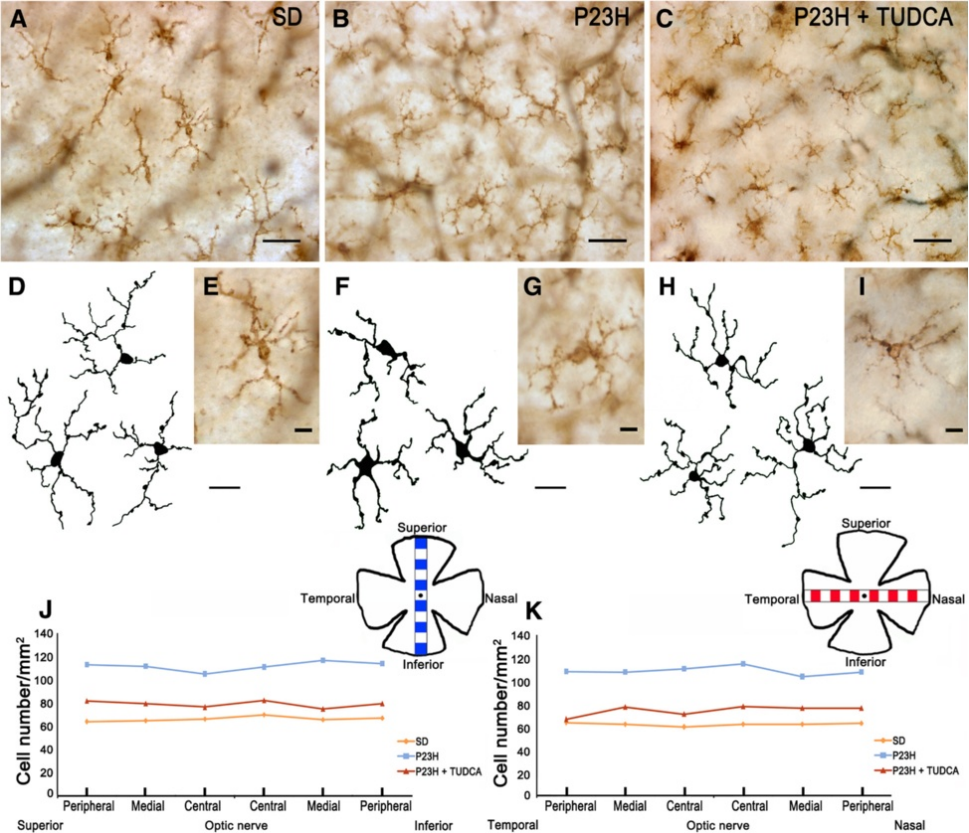
**Morphology, number and distribution of microglial cells in the inner plexiform layer (IPL). (A-** **C)** Representative images of whole-mount rat retinas from a SD **(A)**, untreated P23H **(B)** and tauroursodeoxycholic acid (TUDCA)-treated P23H rat **(C)** labeled with immunoperoxidase. **(E,** **G,** **I)** Magnifications of **(A)**, **(B)** and **(C)**, respectively. **(D**, **F**, **H)** Drawings of microglial cells from SD **(D)**, untreated P23H **(F)** and TUDCA-treated P23H rat **(H)**. **(J, K)** Microglial density, expressed as number of cells per mm^2^, in each of 12 representative regions in each retina: 6 equidistantly arranged on the superior-inferior axis of the retina **(J)** and 6 disposed on the temporal-nasal axis **(K)**. The scheme in the margin of each panel represents the position in the retina of each representative region analyzed. **(A, B, C)** scale bar: 40 μm; **(D, F, H)** scale bar: 20 μm; **(E, G, I)** scale bar: 10 μm.

### TUDCA reduces microglial cell number in P23H rats

To assess the effect of TUDCA on the density and distribution of retinal microglial cells in P23H rats, the relative number of CD11b-positive cells was determined in the GCL, IPL, OPL and SS of whole-mount retinas from SD (n = 4), untreated P23H (n = 6) and TUDCA-treated P23H rats (n = 6). Considering together all sampled areas, untreated P23H rats showed a relative number of immunopositive cells significantly greater compared to normal SD rats (ANOVA, Bonferroni’s test, *P* <0.001; Figure 4A). These differences were significant in the GCL, IPL, OPL and SS (ANOVA, Bonferroni’s test, *P* <0.001 in all cases; Figure 4B). TUDCA-treated P23H rats also showed a mean number of microglial cells per mm^2^ significantly greater than SD rats (ANOVA, Bonferroni’s test, *P* <0.01; Figure 4A), with significant differences in the GCL, IPL and OPL (ANOVA, Bonferroni’s test, *P* <0.05 in GCL and IPL, and *P* <0.001 in OPL; Figure 4B). However, the mean number of CD11b-positive cells was significantly lower in TUDCA-treated P23H rats, as compared to untreated P23H rats (ANOVA, Bonferroni’s test, *P* <0.001; Figure 4A), those differences were statistically significant in the GCL, IPL, OPL and SS (ANOVA, Bonferroni’s test, *P* <0.001 in all cases; Figure 4B). No microglial cells were found in the SS of TUDCA-treated P23H rats, as observed in normal SD rats.

**Figure 8:**
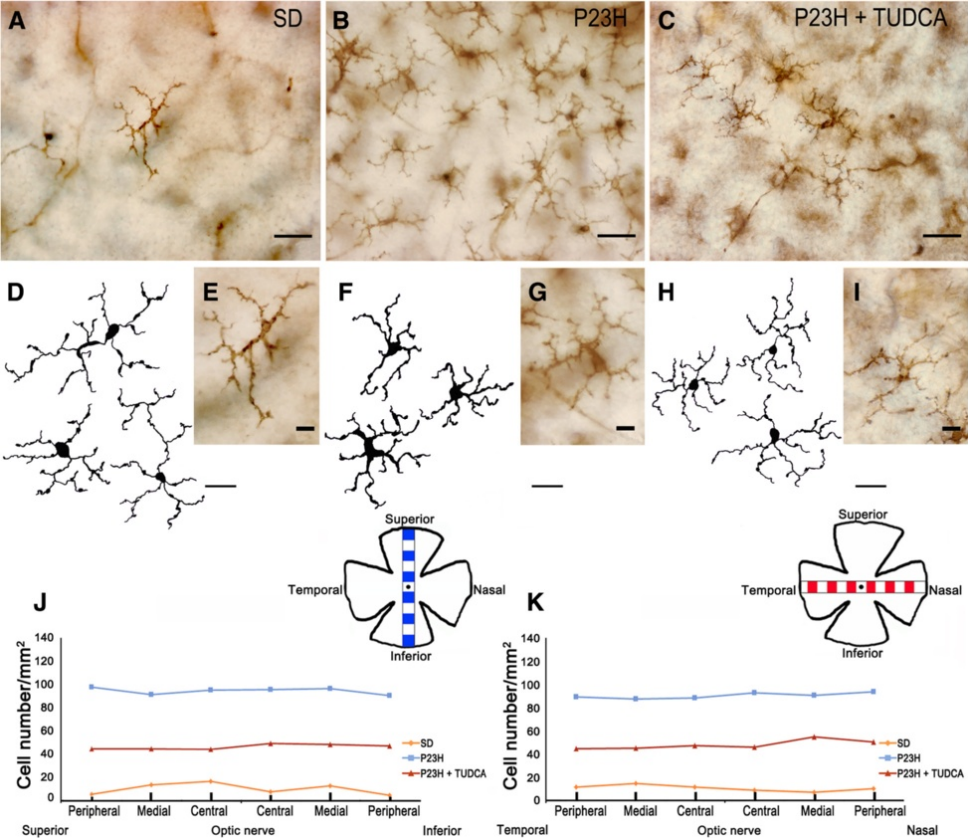
**Morphology, number and distribution of microglial cells in the outer plexiform layer (OPL). (A-** **C)** Representative images of whole-mount rat retinas from a SD **(A)**, untreated P23H **(B)** and tauroursodeoxycholic acid (TUDCA)-treated P23H rat **(C)** labeled with immunoperoxidase. **(E,** **G,** **I)** Magnifications of **(A)**, **(B)** and **(C)**, respectively. **(D,** **F,** **H)** Drawings of microglial cells from SD **(D)**, untreated P23H **(F)** and TUDCA-treated P23H rat **(H)**. **(J, K)** Microglial density, expressed as number of cells per mm^2^, in each of 12 representative regions in each retina: 6 equidistantly arranged on the superior-inferior axis of the retina **(J)** and 6 disposed on the temporal-nasal axis **(K)**. The scheme in the margin of each panel represents the position in the retina of each representative region analyzed. **(A, B, C)** Scale bar: 40 μm; **(D,** **F,** **H)** scale bar: 20 μm; **(E, G, I)** scale bar: 10 μm.

Figure 5 shows representative drawings of the CD11b-positive cells found in the medial area of the retina (superior quadrant) of a SD, untreated P23H and TUDCA-treated P23H rat, differentiating microglial cells located in the GCL, IPL and OPL. According to data obtained in the quantitative analysis shown in Figure 4, we can see that TUDCA-treated P23H rat retinas (Figure 5C) had a density of microglial cells intermediate between the one found in SD rats (Figure 5A) and observed in untreated P23H rats (Figure 5B), indicating a significant effect of TUDCA reducing the relative number of microglial cells in all retinal layers. Representative drawings in Figure 5 (A-C) also show that, regardless of the microglia density, microglial cells are regularly distributed within each retinal layer in SD, untreated P23H and TUDCA-treated P23H rats. As we can see in Figure 5 (D-F) the distribution patterns of microglial cells in the GCL, IPL and OPL, obtained by measuring the distances to the nearest neighbors of each microglial cell, show a Gaussian form and are symmetric around the mean (Figure 5D-F, solid line). As we can see in the figure, these histograms cannot be described by the nearest neighbors analysis of random patterns of the same density and standard deviation (Figure 5D-F, dotted line). It indicates that the distances to the nearest neighbors are uniform, and that microglial cells are arranged in a regular mosaic and are not distributed at random.

**Figure 9:**
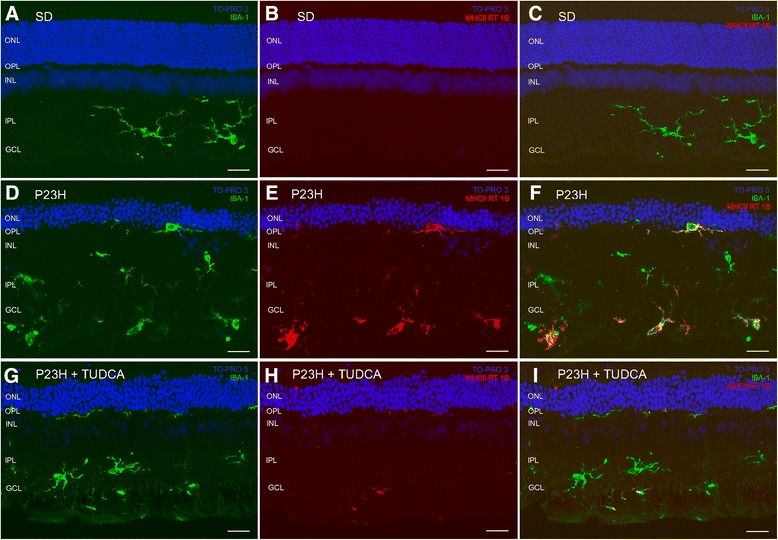
**Activation of microglial cells.** Vertical section of retinas from a SD **(A-** **C)**, untreated P23H **(D-** **F)** and tauroursodeoxycholic acid (TUDCA)-treated P23H **(G-** **I)** rat stained for Iba1 (green; **A**, **D**, **G**) MHC-II RT 1B (red; **B**, **E**, **H**) or both **(C, F, I)**. Nuclei stained with a nuclear marker (TO-PRO 3, blue). All images were collected from the central area of the retina, close to the optic nerve. GCL, ganglion cell layer; IPL, inner plexiform layer; INL, inner nuclear layer; OPL, outer plexiform layer; ONL, outer nuclear layer. Scale bar: 20 μm.

Regardless of the retinal layer in which they were located, most of the microglia found in SD retinas showed a tiny cell soma, little perinuclear cytoplasm, and a large number of fine, branched processes covered in numerous projections. CD11b-positive cells with amoeboid morphology were scarce in SD rats. By contrast, numerous amoeboid microglial cells, with scarce short and thick primary and terminal processes and enlarged soma, were observed in all retinal layers of untreated P23H rats. In TUDCA-treated P23H rats, amoeboid CD11b-positive cells were less abundant than in untreated P23H rats in all layers of the retina. Distribution of microglial cells within each retinal layer (GCL, IPL and OPL; Figures 6, 7 and 8, respectively) was homogeneous in both SD and P23H rats. No differences in microglial density were found between the 12 regions analyzed in each retinal layer. This result implies that the increase in the number of microglial cells in P23H rats compared to SD rats (showed in Figure 4) was analogous in all regions of the GCL, IPL and OPL (Figures 6, 7 and 8, respectively). In TUDCA-treated P23H rats, microglial cells were also homogeneously distributed within the retinal layers, the density of microglial cells in each region of the retina being in these animals lower than that observed in untreated P23H rats.

### TUDCA prevents activation of microglial cells in P23H rats

**Figure 10:**
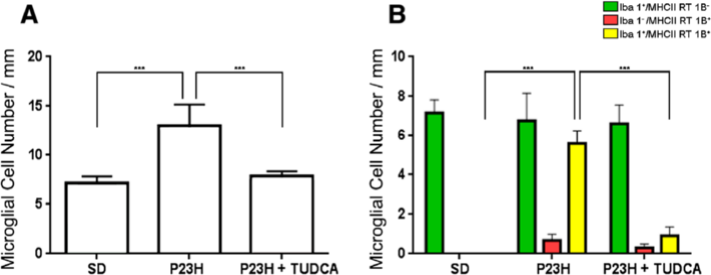
**Quantification of retinal microglial activation. (A)** Average number of Iba1 and/or MHC-II positive microglial cells quantified in vertical retinal sections from SD, untreated P23H and tauroursodeoxycholic acid (TUDCA)-treated P23H rats (6 animals per group). **(B)** Average number of microglial cells showing the expression patterns Iba1^**+**^/MHC-II^**−**^ (green), Iba1^**−**^/MHC-II^**+**^ (red) and Iba1^**+**^/MHC-II^**+**^ (yellow). ***P <0.001; ANOVA, Bonferroni’s test.

Microglial activation was assessed in SD, untreated P23H and TUDCA-treated P23H rats by analyzing vertical retinal sections immunostained with Iba1, a constitutively expressed microglial specific marker, and MHC-II, a marker that is frequently present on activated microglia. The total number of microglial cells expressing one or both markers was counted. As we can see in Figure 9, MHC-II-negative cells labeled with anti-Iba1 antibody (Iba1^**+**^/MHC-II^**−**^) had the typical appearance of resting microglia, while MHC-II-positive cells showed morphologic features of activated microglia; thus proving that MHC-II expression is associated to microglial activation. In SD rat retinas, all Iba1-positive cells found showed morphologic features of resting microglia and were not labeled by anti-MHC-II antibody (Iba1^**+**^/MHC-II^**−**^, Figure 9A-C and Figure 10B). In the retinas of untreated P23H rats, microglia density was significantly higher than the one observed in the SD rats (183%; ANOVA, Bonferroni’s test, *P* <0.001; Figure 9D-E and Figure 10A). Moreover, a high proportion of microglial cells in untreated P23H rat retinas were MHC-II-positive (43%; Figure 10B). Finally, a small group of MHC-II-positive cells found in untreated P23H rat retinas was not labeled by anti-Iba1 antibody (5%; Iba1^**−**^/MHC-II^**+**^; Figure 10B). In TUDCA-treated P23H rats, microglia density was similar to the one observed in the SD rats (108%), and significantly smaller than that found in untreated P23H rats (59%; ANOVA, Bonferroni’s test, *P* <0.001; Figure 9G-I and Figure 10A). The relative number of MHC-II-positive cells not labeled by anti-Iba1 antibody in TUDCA-treated P23H rats was also lower than in untreated P23H rats (27.8%; Iba1^**−**^/MHC-II^**+**^; Figure 10B).

**Figure 11:**
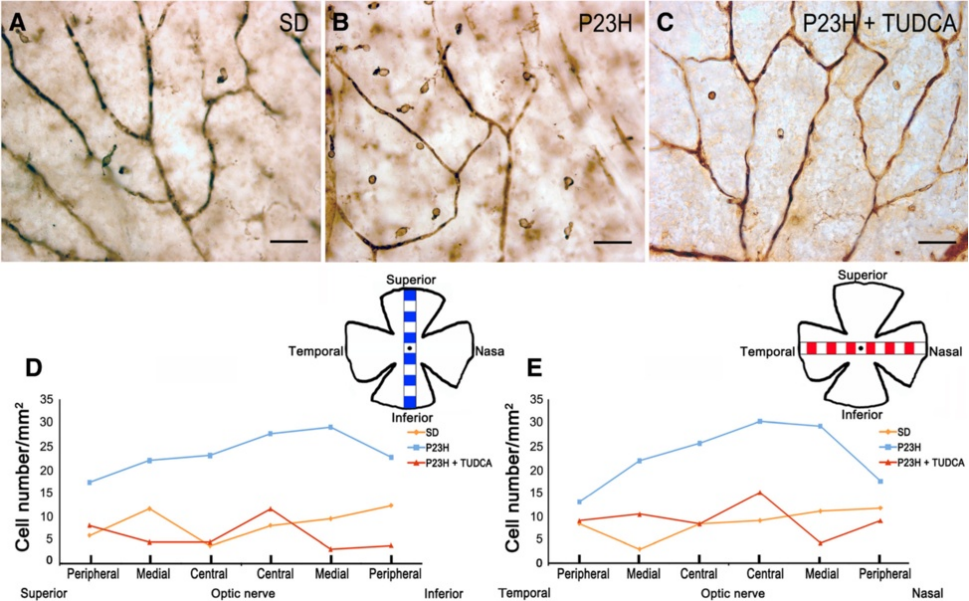
**Number and distribution of macrophages in the ganglion cell layer (GCL). (A-** **C)** Representative images of whole-mount rat retinas from a SD **(A)**, untreated P23H **(B** **)** and tauroursodeoxycholic acid (TUDCA)-treated P23H rat **(C)** labeled with immunoperoxidase. **(D,** **E)** Density of macrophages, expressed as number of cells per mm^2^, in each of 12 representative regions in each retina: 6 equidistantly arranged on the superior-inferior axis of the retina **(D)** and 6 disposed on the temporal-nasal axis **(E)**. The scheme in the margin of each panel represents the position in the retina of each representative region analyzed. Scale bar: 40 μm.

### TUDCA reduces the presence of macrophages in the P23H rats

Immunolabeling of whole-mount retinas with the microglia/macrophages marker CD11b revealed the presence of CD11b-positive cells with morphological characteristics of macrophages in the central, medial and peripheral areas of the GCL in SD, untreated P23H and TUDCA-treated P23H rats (Figure 11). In untreated P23H rats, the mean density of macrophages in the GCL was significantly higher than in SD rats (ANOVA, Bonferroni’s test, *P* <0.001; 23.6 ± 4.8 cells/mm^2^ versus 8.1 ± 1.0 cells/mm^2^; Figure 11). However, TUDCA-treated P23H rats showed a mean density of macrophages in GCL (7.2 ± 1.4 cells/mm^2^) similar to the one observed in SD rats and significantly lower than that found in untreated P23H rats (ANOVA, Bonferroni’s test, *P* <0.001; Figure 11).

## Discussion

The present study revealed that microglia are arranged in regular mosaics homogeneously distributed within the GCL, IPL and OPL in the normal rat retina and degenerative changes in the retina of P23H rats are associated to significant increases in microglia density, as well as to the appearance of a large number of amoeboid CD11b-positive cells, even into the SS. Previous studies have already demonstrated the relation between microglial activation and the progression of neurodegenerative diseases, including RP [18]. However, to our knowledge, this is the first time that changes in microglial cell numbers, distribution and morphology have been described in the P23H rat, a rat model of autosomal dominant RP characterized by a slow-pace retinal degeneration [40,41]. In this study, we also documented that systemic treatment with the antiapoptotic TUDCA attenuates changes in number, distribution and morphologic features of microglial cells in P23H rats.

We identified that in normal Sprague-Dawley rat retinas CD11b-positive microglial cells were homogeneously distributed in the GCL, IPL and OPL; most of them showing morphologic features of resting microglia, although few CD11b-positive cells with amoeboid morphology were found in the GCL. These results agree with previous studies in rats showing that as the layers of the retina differentiate, microglial cells are increasingly restricted to the inner half of the retina [42]. More, recent studies have shown that OX42-immunoreactive microglial cells are distributed mainly in the nerve fiber layer (NFL) and GCL, with some cells localized in the IPL, but rarely in the OPL [43]. On the other hand, previous studies have reported the presence of a network of macrophages in the inner layers of the rat normal retina [44]. In agreement with these previous findings, we found the presence of CD11b-positive cells with morphological characteristics of macrophages in the central, medial and peripheral areas of the GCL in SD rats. The analysis of the distribution pattern of microglial cells in each retinal layer showed that microglial cells are arranged in a regular mosaic in both SD and P23H rats. This result is in accordance with the arrangement in a spatially regular manner of many types of retinal neurons, including photoreceptors [45,46]. The result also suggests the existence of mechanisms involved in the development of regular cellular positioning within each retinal layer.

In untreated P23H rat retinas, microglia density increased in the GCL, IPL and OPL, microglial cells appeared in the SS, and a large number of CD11b-positive cells were labeled with anti-MHC-II, a marker of microglial activation, and showed morphological features of amoeboid cells. The mean density of macrophages in the GCL was also higher in untreated P23H rats as compared to SD rats. These results are in accordance with changes in microglial cells number, activation and distribution previously reported in different forms of disease or retinal damage, like glaucoma [22,47], age-related macular degeneration [20,48], light damage [49,50] and RP [18]. In the mouse model of RP rd10, high levels of pro-inflammatory cytokines and chemokines and early microglial activation have been demonstrated [51,52]. Previous studies have also demonstrated increased density of macrophages after microglial activation [44]. Here we showed that CD11b-positive microglial cells were homogeneously distributed within each of the retinal layers in P23H rats. Nonetheless, retinal degeneration in P23H rats was not homogeneous throughout the retina. Previous studies have reported more advanced stages of degeneration in the medial retina compared with the central and peripheral retina [34,53]. This result evidences that there is no correlation between the state of degeneration in different areas of the retina and glial activation in the same region. The result also suggests that the signals that mediate activation of microglia either do not depend on the state of degeneration of the retina, or are homogeneously distributed within each retinal layer.

Microglial cells have been reported to exhibit the expression of the ionized calcium-binding adaptor molecule 1 (Iba1), a microglia/macrophage-specific calcium-binding protein found in all microglial populations [54-57]. This protein is involved in the membrane ruffling processes of macrophages/microglia so it is considered one of the most important molecules in the motile properties of these cells [54]. In our study, a small group of MHC-II-positive cells was not labeled by anti-Iba1 antibody in P23H rat retinas. This finding can be explained by assuming that microglial cells may have different expression pattern according to their provenance. Perhaps, Iba1^**−**^ cells are migrating microglia that attend to the retinal tissue in response to a chronic negative insult. Another explanation of the presence of Iba1^**−**^ microglial cells could be that these cells are defective or senescent microglial cells that have lost their migrating capacities and down-regulated the Iba1 expression. Possibly, the appearance of this novel expression pattern responds to a different stage of microglial cells within the microglial activation process, wherein each of the stages is characterized by a common expression pattern.

TUDCA in P23H rats reduced the number and activation of microglial cells and macrophages. Moreover, in TUDCA-treated rat retinas microglia were mainly distributed in more internal retinal layers, GCL and IPL, and they were scarce in the OPL and missing in the SS, which is similar to that found in normal SD rat retinas. This result suggests that TUDCA effects on retinal microglia could be due, at least in part, to a reduction in the microglial migratory capacity. Attenuation of microglial activation using TUDCA has been previously demonstrated in experimental models of neuroinflammation, in which it has been reported that TUDCA reduces *in vitro* microglial migration and the expression of chemoattractants required for microglial migration [58]. The effects of TUDCA on retinal microglial cells could be also attributed to an effect of TUDCA on microglial cells behavior, presumably interfering in the respiratory burst of the microglia, which is a critical step in its activation [59,60]. Besides its direct action on microglial cells, TUDCA could act on an indirect way. Through its anti-apoptotic properties, TUDCA slows apoptosis of the retinal tissue [34].

Microglial cells in the retina act as sensors of disarrangement in their micro-environment. Their balanced activities play a key role in the survival of neurons [6,30]. Activation of the microglia has been demonstrated in association with several neurodegenerative diseases, such as Alzheimer’s and Parkinson’s diseases, amyotrophic lateral sclerosis, and multiple sclerosis, although it remains unclear whether microglial activation is a cause or a consequence of neuronal damage [17,61]. The true role of microglia in neurodegenerative diseases, as either a beneficial or harmful factor, still remains controversial, and a large body of results has been obtained to support both hypotheses. Activated microglial cells have been shown to induce photoreceptor death in *in vitro* experiments [62,63], and inhibition of microglial activation reduced photoreceptor apoptosis and significantly improved retinal structure and function [51,64]. In the rd10 mouse model of RP, it has been demonstrated that reductions in the amount of pro-inflammatory cytokines and chemokines by antioxidant treatment inhibits microglial activation and slows the photoreceptor loss [52]. Moreover, it has been demonstrated that microglia are required for the neuroprotective effect of insulin-like growth factor (IGF)-I in the rd10 mouse retina [65]. Hence, the selective inhibition of overacting microglial activity and preservation of their trophic and homeostatic functions appear to be a promising treatment for degenerative diseases [30]. However, it must also be noted that reactive microglial cells can also have a protective effect in damaged retina and that the inhibition of microglial activation can have harmful effects at the same time. In the early stages of the neurodegenerative process, microglial activation can display a protective function through the phagocytosis of cell debris and the release of protective molecules [7,8,17]. In this sense, accumulation of microglia in ischemic areas correlates with a reduction of neuronal damage and confers neuroprotection [66].

## Conclusion

These experiments demonstrate that in normal and disease conditions microglial cells exhibit a mosaic distribution throughout the retinal tissue, independently of the state of degeneration. The data obtained in our work also prove that microglial activation is actively involved in the progression of RP. Moreover, we documented that TUDCA reduces the number and activation of microglial cells in a model of RP (P23H). These results report novel TUDCA anti-inflammatory actions, with potential therapeutic implications for neurodegenerative diseases, including RP. Further studies are required to evaluate the beneficial and/or harmful impact of these effects on retinal structure and function.

## Abbreviations

ABC: avidin-biotin-peroxidase complex
ANOVA: analysis of variance
CNS: central nervous system
DAB: 3,3′-diaminobenzidine tetrahydrochloride
GCL: ganglion cell layer
Iba1: ionized calcium-binding adapter molecule 1
IFN-γ: interferon gamma
IGF: insulin-like growth factor
IL-1α: interleukin 1 alfa
IL-1β: interleukin 1 beta
IL-6: interleukin 6
IPL: inner plexiform layer
NO: nitric oxide
OPL: outer plexiform layer
PB: phosphate buffer
RP: retinitis pigmentosa
RPE: retinal pigment epithelium
RT: room temperature
SD: Sprague Dawley
SEM: standard error of the mean
SS: subretinal space
TNF-α: tumor necrosis factor alfa
TUDCA: tauroursodeoxycholic acid
